# New antibiotics for the treatment of nonfermenting Gram-negative bacteria

**DOI:** 10.1097/QCO.0000000000000757

**Published:** 2021-07-22

**Authors:** Matteo Bassetti, Chiara Russo, Antonio Vena, Daniele Roberto Giacobbe

**Affiliations:** a Department of Health Sciences (DISSAL), University of Genoa; b Clinica Malattie Infettive, San Martino Policlinico Hospital – IRCCS for Oncology and Neurosciences, Genoa, Italy

**Keywords:** cefiderocol, ceftazidime/avibactam, ceftolozane/tazobactam, eravacycline, imipenem/relebactam, *Pseudomonas acinetobacter*

## Abstract

To discuss the current literature on novel agents for the treatment of carbapenem-resistant nonfermenting Gram-negative bacteria (NF-GNB) infections.

**Recent findings:**

Some novel agents have recently become available that are expected to replace classical polymyxins as the first-line options for the treatment of carbapenem-resistant NF-GNB infections.

**Summary:**

In this narrative review, we provide a brief overview of the differential activity of various recently approved agents against NF-GNB most encountered in the daily clinical practice, as well as the results from phase-3 randomized clinical trials and large postapproval observational studies, with special focus on NF-GNB. Since resistance to novel agents has already been reported, the use of novel agents needs to be optimized, based on their differential activity (not only in terms of targeted bacteria, but also of resistance determinants), the local microbiological epidemiology, and the most updated pharmacokinetic/pharmacodynamic data. Large real-life experiences remain of crucial importance for further refining the optimal treatment of NF-GNB infections in the daily clinical practice.

## INTRODUCTION

Nonfermenting Gram-negative bacteria (NF-GNB) are ubiquitous, aerobic, nonspore forming bacilli, including some organisms that may show concomitant resistance to several classes of antibacterials commonly used in human medicine [1,2]. NF-GNB may be responsible for healthcare-associated infections caused by strains resistant to all classical beta-lactams (including carbapenems), fluoroquinolones, and aminoglycosides, leaving clinicians with very few, and sometimes none, therapeutic options when facing NF-GNB infections in hospitalized patients [3,4,5^▪^].

In the last few decades, the treatment of severe healthcare-associated NF-GNB infections has been mostly based on polymyxin monotherapy or polymyxin-based combined regimens [6,7]. Nonetheless, although classical polymyxins (colistin and polymyxin B) have undoubtedly long represented an important first-line resource for curtailing mortality of NF-GNB infections, it has also been widely recognized now that they have some peculiar pharmacokinetic/pharmacodynamic (PK/PD) characteristics possibly resulting in impaired efficacy in comparison with other active therapeutic options, when the latter are available [8,9^▪^,^10^]. Consequently, although colistin and polymyxin B certainly remain a crucial resource for salvage therapy or when there are no other active options, some other novel agents have recently become available (or will become available in the forthcoming future) that are expected to replace classical polymyxins as the first-line options for the treatment of carbapenem-resistant NF-GNB infections.

In this narrative review, we briefly discuss the current literature on recently approved novel agents for the treatment of carbapenem-resistant NF-GNB infections. 

**Box 1:**
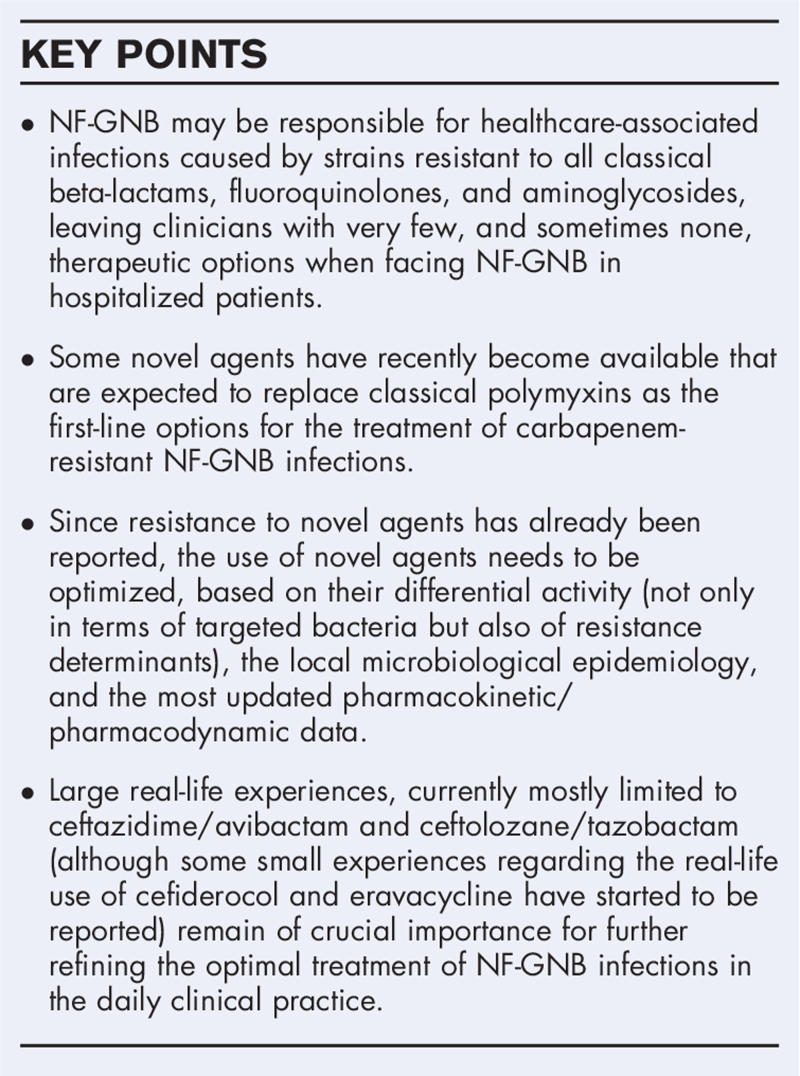
no caption available

### Recently approved agents with activity against carbapenem-resistant nonfermenting Gram-negative bacteria

The following paragraphs provide a brief overview of the differential activity of various recently approved agents against NF-GNB most encountered in the daily clinical practice, as well as a list of currently approved indications and the results from phase-3 randomized clinical trials (RCTs) with focus on NF-GNB. A summary of large postapproval observational studies on the use of novel agents for the treatment of NF-GNB infections in real-life is provided in Table 1.

**Table 1:** Observational studies on the real-life use of novel agents for the treatment of NF-GNB infections in adult patients^∗^

Study, year [Ref]	Study design	Population	Type of NF-GNB infection/s	Results
Balandin *et al.*, 2021 [90]	• Multicenter• Retrospective	• 95 critically ill patients receiving ≥48 h of ceftolozane/tazobactam for *Pseudomonas aeruginosa* infections	• Most *P. aeruginosa* infections were nosocomial pneumonia (54/95, 56.8%)	• A favorable response was registered in 68/95 patients (71.6%)• 30-day mortality was 30.5% (29/95)
Bassetti *et al.*, 2019 [91]	• Multicenter• Retrospective	• 101 hospitalized patients receiving ≥96 h of ceftolozane/tazobactam for *P. aeruginosa* infections	• Most *P. aeruginosa* infections were nosocomial pneumonia (32/101, 31.7%) followed by ABSSSI (21/101, 20.8%)	• A successful clinical outcome was registered in 84/101 patients (83.2%)• Five deaths were registered
Castan *et al.*, 2021 [92]	• Multicenter• Prospective	• 84 hospitalized patients receiving at least one dose of ceftolozane/tazobactam. Mostly for pneumonia (42/84, 50.0%)	• 71/84 patients (84.5%) had *Pseudomonas aeruginosa* infection (mostly nosocomial pneumonia, 18/33, 54.5%)	• Outcome data available for 72/84 patients (85.7%) and not stratified for causative agent• Clinical cure was registered in 44/72 patients (61.1%)• Mortality was 5.6% (4/72)
Cultrera *et al.*, 2020 [93]	• Single center• Retrospective	• 122 patients receiving ≥72 h of ceftolozane/tazobactam for GNB infections	• 69/241 isolates (28.6%) from 122 patients treated with ceftolozane/tazobactam were *P. aeruginosa*	• Microbiological cure was observed in 99/122 included patients (81%), no numerator and denominator available for the subgroup of *P. aeruginosa* infections
Díaz-Cañestro *et al.*, 2018 [94]	• Single center• Prospective	• 58 hospitalized patients receiving ceftolozane/tazobactam for *P. aeruginosa* infections	• Most were *P. aeruginosa* respiratory tract infections (35/58, 60.3%)	• Clinical cure was registered in 37/58 patients (63.8%)• 28-day mortality was 27.6% (16/58)
Escolà-Vergé *et al.*, 2018 [95]	• Single center• Retrospective	• 38 hospitalized patients receiving ceftolozane/tazobactam for *P. aeruginosa* infections	• Most were *P. aeruginosa* respiratory tract infections (14/38, 36.8%)	• Clinical cure at the end of treatment was registered in 33/38 patients (86.8%)• All-cause mortality was 13.2% (5/38)
Gallagher *et al.*, 2018 [96]	• Multicenter• Retrospective	• 205 hospitalized patients receiving ≥24 h of ceftolozane/tazobactam for *P. aeruginosa* infections	• Most *P. aeruginosa* infections were pneumonia (121/205, 59.0%)	• Clinical success was registered in 151/205 patients (73.7%)• Microbiological cure was registered in 145/205 patients (70.7%)• Mortality was 19.0% (39/205)
Haidar *et al.*, 2017 [97]	• Single center• Retrospective	• 21 hospitalized patients receiving ceftolozane/tazobactam for *P. aeruginosa* infections	• Most *P. aeruginosa* infections were respiratory tract infections (18/21, 85.7%)	• Clinical success was registered in 15/21 patients (71.4%)• 30-day all-cause mortality was 9.5% (2/21)
Hart *et al.*, 2021 [98]	• Multicenter• Retrospective	• 69 immunocompromised patients receiving ≥24 h of ceftolozane/tazobactam for *P. aeruginosa* infections	• Most *P. aeruginosa* infections were respiratory tract infections (39/69, 56.5%)	• Clinical cure was registered in 47/69 patients (68.1%)• 30-day all-cause mortality was 18.8% (13/69)
Jorgensen *et al.*, 2019 [99]	• Multicenter• Retrospective	• 203 hospitalized patients receiving ≥72 h of ceftazidime/avibactam	• 63/203 patients (31.0%) had *Pseudomonas aeruginosa* infection (mostly nosocomial pneumonia, 38/63, 60.3%)	• Clinical success was registered in 44/63 patients (69.8%)• 30-day all-cause mortality was 17.5% (11/63)
Jorgensen *et al.*, 2020 [100]	• Multicenter• Retrospective	• 259 hospitalized patients receiving ≥72 h of ceftolozane/tazobactam	• 226/259 patients (87.3%) had multidrug-resistant *P. aeruginosa* infection (mostly nosocomial pneumonia, 149/226, 65.9%)	• Clinical success was registered in 141/226 patients (62.4%)• 30-day all-cause mortality was 17.3% (39/226)
Munita *et al.*, 2017 [101]	• Multicenter• Retrospective	• 35 hospitalized patients receiving ≥72 h of ceftolozane/tazobactam for *P. aeruginosa* infections	• Most infections were *P. aeruginosa* pneumonia (18/35, 51.4%)	• Treatment success was registered in 26/35 patients (74.3%)
Pogue *et al.*, 2020 [102]	• Multicenter• Retrospective	• 200 hospitalized patients receiving ceftolozane/tazobactam or polymyxin-based regimens or aminoglycosides-based regimens for *P. aeruginosa* infections	• 100 patients with *P. aeruginosa* infections received ceftolozane/tazobactam, mostly for ventilator-associated pneumonia (52/100, 52.0%)	• Clinical cure was registered in 81/100 (81.0%) and 61/100 (61.0%) of patients receiving ceftolozane/tazobactam and polymyxin/ aminoglycosides-based regimens, respectively• In-hospital mortality was 20.0% (20/100) and 25.0% (25/100) in patients receiving ceftolozane/tazobactam and polymyxin/ aminoglycosides-based regimens, respectively
Rodríguez-Núñez *et al.*, 2019 [103]	• Multicenter• Retrospective	• 90 hospitalized patients receiving ≥72 h of ceftolozane/tazobactam for *P. aeruginosa* lower respiratory tract infections	• 63/90 (70.0%) patients had pneumonia, and 27/90 (30.0%) had tracheobronchitis	• Clinical success was registered in 51/90 patients (56.7%)• 30-day all-cause mortality was 27.8% (25/90)
Sacha *et al.*, 2017 [104]	• Single center• Retrospective	• 60 hospitalized patients receiving ceftolozane/tazobactam, mostly for pneumonia (34/60, 56.7%)	• 52/60 patients (86.7%) had *P. aeruginosa* infection (mostly nosocomial pneumonia, 149/226, 65.9%)	• Clinical cure was registered in 25/39 patients with ceftolozane/tazobactam-susceptible *P. aeruginosa* infections (64.1%)
Vena *et al.*, 2020 [105]	• Multicenter• Retrospective	• 41 hospitalized patients receiving ≥72 h of ceftazidime-avibactam for GNB other than carbapenem-resistant Enterobacterales	• 33/41 patients (80.5%) had *P. aeruginosa* infection (mostly nosocomial pneumonia, 18/33, 54.5%)	• Clinical cure was registered in 29/33 patients with *P. aeruginosa* infection• One death was registered
Xipell *et al.*, 2018 [106]	• Single center• Retrospective	• 23 hospitalized patients with 24 episodes of *P. aeruginosa* infections treated with ceftolozane/tazobactam	• Clinical cure was registered in 29/33 patients with *P. aeruginosa* infection• One death was registered	• Clinical cure was registered in 21/24 episodes (87.5%)• 6-week mortality was 22% (5/23 patients)
Zhanel *et al.*, 2021 [107]	• Multicenter• Prospective	• 51 hospitalized patients receiving ceftolozane/tazobactam, mostly for respiratory tract infections (32/51, 62.7%)	• Ceftolozane/tazobactam was used as directed therapy for *P. aeruginosa* infections in 92.2% of patients (47/51)	• Clinical and microbiological successes of 64.4% and 60.5% were reported, respectively• Seven deaths were registered

ABSSSI, acute bacterial skin and skin structure infections; NF-GNB, nonfermenting Gram-negative bacteria.

a Only studies including at least 20 patients receiving a specific novel agent for a specific member of NF-GNB are reported in the table.

### Ceftazidime/avibactam

Ceftazidime/avibactam is a novel β-lactam/β-lactamase inhibitor (BL/BLI) combination of a well-known third-generation cephalosporin with the diazabicyclooctane non-BL, BLI avibactam [11,12]. Avibactam can inhibit class A (e.g., KPC-type) and some class D (e.g., some OXA-type) but not class B (metallo-β-lactamases [MBLs]) carbapenemases [12]. For this reason, one may erroneously expect inactivity of ceftazidime/avibactam against carbapenem-resistant *Pseudomonas aeruginosa* (CRPA), since *P. aeruginosa* may express MBLs, and activity against carbapenem-resistant *Acinetobacter baumannii* (CRAB), that may show resistance to carbapenems due to the expression of OXA-type carbapenemases [13^▪^,^14^]. However, the true picture is more complex, with *in vitro* studies demonstrating a somewhat opposite reality: activity against several CRPA isolates (albeit not against MBLs producers) and inactivity against CRAB [15–17]. The reasons rely on ceftazidime/avibactam activity against a nonnegligible proportion of noncarbapenemase-producing CRPA (especially in the case of derepressed AmpC) and on its inefficient hydrolysis of the OXA-type carbapenemases harbored by CRAB [18–23]. *In vitro* activity against *Stenotrophomonas maltophilia* and *Burkholderia cepacia* complex has also been reported [24–26]. Based on the results of different phase-3 RCTs [27–30], the US Food and Drug Administration (FDA) and the European Medicines Agency (EMA) have approved ceftazidime/avibactam for the treatment of complicated urinary tract infections (cUTI), complicated intraabdominal infections (cIAI), hospital-acquired bacterial pneumonia (HABP), and ventilator-associated bacterial pneumonia (VABP). EMA has also approved ceftazidime/avibactam for the treatment of aerobic GNB infections with limited treatment options in adult patients. It is of note that in phase 3 RCTs *P. aeruginosa* was isolated in ≤10% of patients with microbiological diagnosis, except for the REPROVE RCT, that evaluated the efficacy of ceftazidime/avibactam vs. meropenem for the treatment of HABP and VABP, and in which *P. aeruginosa* represented 30% of isolates in the microbiologically modified intention-to-treat population [29]. It is worth noting that in this trial, which overall demonstrated noninferiority of ceftazidime/avibactam vs. meropenem, ceftazidime/avibactam was evaluated as an alternative to carbapenem and not as a treatment for HABP and VABP caused by carbapenem-resistant organisms. Given this necessary premise, the rates of clinical cure in patients with *P. aeruginosa* infection in ceftazidime/avibactam and meropenem arms were 64% (27/42) and 77% (27/35), respectively (difference −12.8%, with 95% confidence interval [CI] from −32.3 to 8.0), whereas the rates of favorable microbiological response were 43% (18/42) and 40% (14/35), respectively (difference 2.9%, with 95% CI from −19.1 to 24.3) [29].

### Ceftolozane/tazobactam

Ceftolozane/tazobactam is the combination of a broad-spectrum oxyimino-cephalosporin and the BLI tazobactam, with ceftolozane showing broader activity than ceftazidime against essential penicillin-binding proteins (PBPs) of *P. aeruginosa* such as PBP1b, PBP1c, PBP2, and PBP3 [31,32]. On the other hand, ceftolozane exhibits lower affinity than imipenem for PBP4, thereby avoiding induction of AmpC overexpression [32]. Ceftolozane is also stable against chromosomal AmpC of *P. aeruginosa*[31,33]. Finally, ceftolozane/tazobactam is a poor substrate of *P. aeruginosa* Mex efflux pumps and its entry in the bacterial cell is not affected by mutations of the OprD porin [34,35]. All of this may explain why potent *in vitro* activity against CRPA was detected in many studies, despite resistance was observed in the case of carbapenemases production [36–43]. *In vitro* studies have also registered some activity of ceftolozane/tazobactam against *S. maltophilia* and *B. cepacia* complex, albeit any possible clinical implication of these findings deserves further investigation [44,45]. The FDA and EMA have approved ceftolozane/tazobactam for the treatment of cUTI, cIAI, HABP, and VABP. Regarding per-pathogen subgroup analyses in phase 3 RCTs, in the ASPECT-cIAI RCT (demonstrating noninferiority of ceftolozane/tazobactam plus metronidazole vs. meropenem for the treatment of cIAI) clinical cure in patients with cIAI due to *P. aeruginosa* was 100% (26/26) and 93% (27/29) in ceftolozane/tazobactam plus metronidazole arm and in meropenem arm, respectively [46]. In the ASPECT-cUTI RCT (showing noninferiority of ceftolozane/tazobactam vs. levofloxacin for the treatment of cUTI) microbiological eradication in patients with cUTI due to *P. aeruginosa* was 86% (6/7) and 58% (7/12) in ceftolozane/tazobactam and levofloxacin arms, respectively [47]. In the ASPECT-NP RCT (showing noninferiority of ceftolozane/tazobactam vs. meropenem for the treatment of HABP and VABP), clinical response in patients with *P. aeruginosa* pneumonia was registered in 57% (36/63) and 60% (39/65) of patients in ceftolozane/tazobactam and meropenem arms, respectively (difference −2.9%, with 95% CI from -19.4 to 13.8), whereas the 28-day all-cause mortality in patients with *P. aeruginosa* pneumonia was 25.4% (16/63) and 18.5% (12/65) in ceftolozane/tazobactam and meropenem arms, respectively (difference −6.9 with 95% CI from −21.1 to 7.42) [48]. As for ceftazidime/avibactam, in phase 3 RCTs ceftolozane/tazobactam was not specifically investigated for the treatment of carbapenem-resistant infections, whereas many large observational experiences are available (see Table 1).

### Imipenem/relebactam

Imipenem/relebactam combines imipenem with relebactam, a diazabicyclooctane non-BL, BLI able to inhibit class A, but not class B and class D carbapenemases [4,49]. Although this is in line with imipenem/relebactam inactivity against CRAB and MBL-producing CRPA, activity against noncarbapenemase producing CRPA may be retained owing to its activity against CRPA strains with OprD porin loss combined with AmpC overexpression, as well as to the fact that neither imipenem nor relebactam is affected by the MexAB-OprM efflux pump [50–54]. Intrinsic resistance of *S. maltophilia* and *B. cepacia* complex to imipenem and reduced activity against *A. baumannii* may preclude the use of imipenem-relebactam for the treatment of infections caused by these NF-GNB [55–57]. The FDA has approved imipenem/relebactam for the treatment of cUTI, cIAI, HABP, and VABP, whereas EMA has also approved it for the treatment of aerobic GNB infections with limited treatment options in adult patients. In the RESTORE-IMI 2 RCT (demonstrating noninferiority of imipenem/relebactam vs. piperacillin/tazobactam for the treatment of HABP and VABP), a favorable clinical response in patients with *P. aeruginosa* pneumonia was registered in 47% (7/15) and 68% (17/25) of patients in imipenem/relebactam and piperacillin/tazobactam arms, respectively (difference −21.3%, with 95% CI from −49.7 to 10.0), whereas 28-day all-cause mortality in patients with *P. aeruginosa* pneumonia was 33.3% (5/15) and 12.0% (3/25) in imipenem/relebactam and piperacillin/tazobactam arms, respectively (difference 21.3%, with 95% CI from −4.5 to 48.9) [58]. The RESTORE-IMI 1 RCT was a double-blind RCT comparing imipenem-relebactam vs. colistin plus imipenem for the treatment of cUTI, cIAI, HABP, or VABP caused by imipenem-resistant bacteria, that in 77% of cases were CRPA [59]. A favorable overall response (primary endpoint, defined as 28-day all-cause mortality for HABP/VAP, clinical response for cIAI, and a composite of clinical response and microbiological response for cUTI) in patients with CRPA was 81% (13/16) and 63% (5/8) in imipenem/relebactam arm and colistin plus imipenem arm, respectively. Notably, treatment-emergent nephrotoxicity was overall registered in 10% (3/29) and 56% (9/16) of patients in imipenem/relebactam arm and colistin plus imipenem arm, respectively (difference −45.9%, with 95% CI from −69.1 to −18.4) [59].

### Cefiderocol

Cefiderocol is a siderophore cephalosporin that exploits the iron transporters of the outer membrane of GNB, thereby reaching the periplasmic space and avoiding possible resistance mechanisms such as porin loss or efflux pumps [60–63]. Regarding β-lactamases, cefiderocol has shown potent *in vitro* activity against GNB harboring all classes of carbapenemases (A, B, and D), including MBL-producing CRPA and OXA-type carbapenemase-producing CRAB [64–67]. Furthermore, *in vitro* activity of cefiderocol against *S. maltophilia* and *B. cepacia* complex has also been reported [64–66,68,69]. The FDA has approved cefiderocol for the treatment of cUTI, HABP, and VABP, whereas EMA has approved it for the treatment of aerobic GNB infections with limited treatment options in adult patients. The CREDIBLE-CR study was an open-label, phase 3 RCT comparing cefiderocol vs. best available therapy (mostly colistin-based regimens) for the treatment of bloodstream infections (BSI), cUTI, healthcare-associated bacterial pneumonia (HCABP), HABP, and VABP caused by carbapenem-resistant GNB [70]. Overall, clinical cure rates were similar in the two arms, being 52.5% (42/80) and 50% (19/38) in cefiderocol and best available therapy arms, respectively. On the other hand, an imbalance in mortality, which was the largest for 49-day mortality, was registered (34% vs. 18% in cefiderocol and best available therapy arms, respectively). This imbalance was more marked for CRAB infections, with a registered 49-day mortality of 50% (21/42) and 18% (3/17) in cefiderocol and best available therapy arms, respectively. The reasons for this imbalance are still partly unknown (some possibilities are chance alone related to the small sample size, the increased prevalence of some baseline variables possibly indicating more severe disease in patients with CRAB infections treated with cefiderocol than in those treated with best available therapy, or an actual reduced efficacy of cefiderocol for CRAB infections deserving further investigation). Of note, all-cause mortality in patients with CRPA infection (and without concomitant CRAB infection) was 18% (2/11) in both arms [70]. In the APEKS-NP RCT (demonstrating noninferiority of cefiderocol vs. meropenem for the treatment of HABP and VABP) the 14-day all-cause mortality in patients with *P. aeruginosa* pneumonia was 8% (2/24) and 13% (3/23) in cefiderocol and meropenem arms, respectively (difference −4.7%, with 95% CI from −22.4 to 12.9), whereas the 14-day all-cause mortality in patients with *A. baumannii* pneumonia was 22% (5/23) and 17% (4/24) in cefiderocol and meropenem arms, respectively (difference 5.1%, with 95% CI from −17.4 to 27.6) [71]. In the APEKS-cUTI RCT (demonstrating noninferiority of cefiderocol vs. imipenem-cilastatin for the treatment of cUTI) the composite endpoint of clinical and microbiological cure at test of cure in patients with cUTI due to *P. aeruginosa* was achieved in 47% (7/15) and 50% (2/4) of patients in cefiderocol and imipenem-cilastatin arms, respectively [72].

### Eravacycline

Eravacycline is a novel synthetic fluorocycline that can elude some resistance mechanisms affecting tetracyclines such as efflux pumps and ribosomal protection [73,74]. Eravacycline is inactive against *P. aeruginosa*, whereas *in vitro* activity has been registered against CRAB [73–75]. Eravacycline has also been shown to be active *in vitro* against *S. maltophilia*, but not against *B. cepacia* complex [76,77]. Eravacycline has been approved by the FDA and EMA for the treatment of cIAI. In the IGNITE 1 RCT (showing noninferiority of eravacycline vs. ertapenem for the treatment of cIAI requiring surgical or percutaneous intervention), clinical cure in patients with cIAI due to *P. aeruginosa* was registered in 83% (15/18) and 90% (18/20) of patients in eravacycline and ertapenem arms, respectively, whereas in patients with cIAI due to *Acinetobacter* spp. clinical cure was registered in 100% (8/8) and 100% (6/6) of patients in eravacycline and ertapenem arms, respectively [78]. In the IGNITE 4 RCT (showing noninferiority of eravacycline vs. meropenem for the treatment of cIAI), clinical cure in patients with cIAI due to *P. aeruginosa* was registered in 95% (18/19) and 90% (18/20) of patients in eravacycline and meropenem arms, respectively, whereas in patients with cIAI due to *Acinetobacter baumannii*, clinical cure was registered in 100% (5/5) and 100% (2/2) of patients in eravacycline and meropenem arms, respectively [79].

## CONCLUSION

**FIGURE 1:**
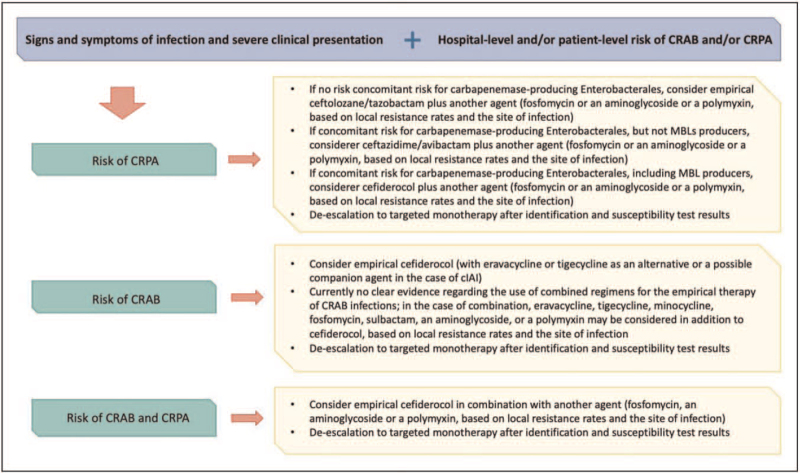
Proposed algorithm for the empirical use of novel agents in patients with suspected NF-GNB infections. cIAI, complicated intra-abdominal infections. CRAB, carbapenem-resistant *Acinetobacter baumannii*; CRPA, carbapenem-resistant *Pseudomonas aeruginosa*; MBLs, metallo-β-lactamases; NF-GNB, nonfermenting Gram-negative bacteria.

We are entering a novel chapter in the treatment of NF-GNB infections, in which novel β-lactams and β-lactams/β-lactamases inhibitor combinations allow a renewed activity of these classes of antibacterials against CRPA and CRAB. Furthermore, sulbactam/durlobactam (active against CRAB) is in phase 3 of clinical development and other agents showing *in vitro* activity against CRAB and/or CRPA have reached clinical development [80,81^▪^]. All of this could reasonably imply the replacement of polymyxin-based regimens as first-line treatment on most occasions, both for empirical and targeted therapy (a possible algorithm for empirical treatment is shown in Fig. 1). Nonetheless, since resistance to novel agents has also and already been reported [31,82,83], their use need to be optimized, based on their differential activity (not only in terms of targeted bacteria but also of resistance determinants [84,85^▪^]), the most updated PK/PD data, and the local microbiological epidemiology. Notably, large real-life experiences, that currently are mainly limited to ceftazidime/avibactam and ceftolozane/tazobactam (although some small experiences regarding the real-life use of cefiderocol and eravacycline have also started to be reported [86–89]), remain of crucial importance for further refining the optimal treatment of NF-GNB infections in the daily clinical practice.

## Acknowledgements

*None.*

### Financial support and sponsorship

*None.*

### Conflicts of interest

*Outside the submitted work, M.B. has received funding for scientific advisory boards, travel, and speaker honoraria from Angelini, Astellas, AstraZeneca, Basilea, Bayer, BioMérieux, Cidara, Correvio, Cubist, Menarini, Molteni, MSD, Nabriva, Paratek, Pfizer, Roche, Shionogi, Tetraphase, Thermo Fisher, and The Medicine Company. Outside the submitted work, D.R.G. reports honoraria from Stepstone Pharma GmbH, unconditional grants from MSD Italia and Correvio Italia, and grants to his institution for investigator-initiated research from Pfizer Inc. The other authors have no conflicts of interest to declare.*

*Funding: none*

## REFERENCES AND RECOMMENDED READING

Papers of particular interest, published within the annual period of review, have been highlighted as:

▪ of special interest
▪▪ of outstanding interest
